# A Comparative Study of the Metabolic and Skeletal Response of C57BL/6J and C57BL/6N Mice in a Diet-Induced Model of Type 2 Diabetes

**DOI:** 10.1155/2015/758080

**Published:** 2015-06-03

**Authors:** Elizabeth Rendina-Ruedy, Kelsey D. Hembree, Angela Sasaki, McKale R. Davis, Stan A. Lightfoot, Stephen L. Clarke, Edralin A. Lucas, Brenda J. Smith

**Affiliations:** ^1^Department of Nutritional Sciences, Oklahoma State University, Stillwater, OK 74078, USA; ^2^Center for Cancer Prevention and Drug Development, University of Oklahoma Health Sciences Center, Oklahoma City, OK 73104, USA

## Abstract

Type 2 diabetes mellitus (T2DM) represents a complex clinical scenario of altered energy metabolism and increased fracture incidence. The C57BL/6 mouse model of diet-induced obesity has been used to study the mechanisms by which altered glucose homeostasis affects bone mass and quality, but genetic variations in substrains of C57BL/6 may have confounded data interpretation. This study investigated the long-term metabolic and skeletal consequences of two commonly used C57BL/6 substrains to a high fat (HF) diet. Male C57BL/6J, C57BL/6N, and the negative control strain, C3H/HeJ, mice were fed a control or HF diet for 24 wks. C57BL/6N mice on a HF diet demonstrated an increase in plasma insulin and blood glucose as early as 4 wk, whereas these responses were delayed in the C57BL/6J mice. The C57BL/6N mice exhibited more severe hepatic steatosis and inflammation. Only the C57BL/6N mice lost significant trabecular bone in response to the high fat diet. The C3H/HeJ mice were protected from bone loss. The data show that C57BL/6J and C57BL/6N mice differ in their metabolic and skeletal response when fed a HF diet. These substrain differences should be considered when designing experiments and are likely to have implications on data interpretation and reproducibility.

## 1. Introduction

Increasing prevalence of type 2 diabetes mellitus (T2DM) has stimulated research focused on the pathogenesis and treatment of T2DM and its complications. Initial studies examining fracture as a possible complication of T2DM indicated that type 2 diabetics were not at risk of fracture based on bone mineral density (BMD), the clinical standard for screening [1–3]. However, data analyzed from clinical trials with fracture as an outcome variable instead of BMD revealed that both men and women with T2DM experience an increase in fracture (i.e., 1.5–3-fold) beginning 5–10 years after diagnosis [4–8]. Collectively, the clinical evidence indicates that, independent of BMD, type 2 diabetics are at increased risk of fracture that is exacerbated over time.

Rodent models have enabled investigators to study the molecular mechanisms involved in altering bone quality in T2DM [9]. One of the most commonly utilized models has been the C57BL/6 young growing mouse fed a diet high in total and saturated fat (HF), which exhibits an increase in adiposity, impaired glucose tolerance, and dyslipidemia, similar to prediabetes in humans [10–12]. C57BL/6 mice have been reported to exhibit decreased trabecular bone and either increased, decreased, or no change in cortical bone in response to long-term intake of a HF diet [13–17]. When alterations in bone microarchitecture occur, they are usually accompanied by impaired bone quality as evidenced by compromised biomechanical properties [15, 17–21]. Factors that may contribute to some of the discrepancies in the literature describing the skeletal response to a HF diet could be due to the composition of the diets, the age of the mice, the duration of the study, and, importantly, differences in the C57BL/6 mouse substrain's response (e.g., C57BL/6J or C57BL/6N) [13–16, 21].

A review of published reports revealed that studies utilizing different C57BL/6 substrains (e.g., C57BL/6J and C57BL/6N) are often treated interchangeably without mention of genetic variations that could have important implications on the results and their interpretation. For example, the C57BL/6J mouse has a missense mutation in the gene encoding nicotinamide nucleotide transhydrogenase (*Nnt*) that alters RNA splicing and leads to the deletion of exons 7–11 [22–24]. When C57BL/6J mice are fed a high fat diet, they exhibit impaired glucose tolerance that appears to result from suppressed insulin secretion by pancreatic *β*-cells [25]. Thus, genetic differences in the C57BL/6J mouse could contribute to some of the discrepancies in the literature in regard to metabolic and skeletal responses reported in the diet-induced obesity model of T2DM.

In contrast to the C57BL/6 mouse, the C3H/HeJ mouse model has been used in mechanistic studies because of its blunted metabolic response to a high fat diet [26, 27]. C3H/HeJ mice have a nonfunctional toll-like receptor (TLR) 4 due to a point mutation in the toll-interleukin 1 receptor domain [26–28]. TLR-4 is expressed on bone cells (i.e., both the osteoblasts and osteoclasts) [29, 30]. Our lab and others [31–34] have shown that TLR-4 ligands (e.g., lipopolysaccharide or LPS and saturated free fatty acids or sFFAs) as well as downstream inflammatory mediators have the potential to uncouple bone turnover. Because of interest in sFFAs and gut-derived LPS in the pathophysiology of T2DM and its complications, the C3H/HeJ strain has become an important research tool to examine the role of TLR-4 in these metabolic responses.

To date, a direct comparison of the long-term metabolic and skeletal responses of the C57BL/6J and C57BL/6N substrains to a HF diet has not been reported in the literature. If, as we hypothesized, the metabolic response to a HF diet in these two substrains differs due to genetic variations, this may alter the inflammatory response, hormones, and adipokines, subsequently affecting the bone. Such differences would be important relative to the interpretation of results and could assist investigators in selecting the most appropriate model. Furthermore, because of our laboratory's interest in TLR-4 and bone, in this study C3H/HeJ mice were used as a negative control for comparative purposes [26, 27].

## 2. Methods

### 2.1. Animal Care and Diets

Eight-week-old male mice, C57BL/6N from Charles River (Wilmington, MA) and C57BL/6J and C3H/HeJ mice from Jackson Labs (Bar Harbor, ME), were obtained (*n* = 30 mice/strain) for these studies. Animals were acclimated for 7 days and then randomly assigned to a control AIN-93M (10% kcals from fat) or a HF (45% kcals from fat; Harlan Teklad, TD.06415) diet for 24 wk. Body weight and food intake were recorded throughout the study. Total feed efficiency was calculated by determining the gain in body weight (mg) per energy unit consumed (kcal) [35]. Venous tail blood was collected following a 6 hr fast for evaluation of glucose and insulin at 4 wk intervals. After 24 wk, mice were anesthetized (ketamine/xylazine cocktail 70 and 30 mg/kg body weight, resp.) as previously reported and whole body DXA (Lunar PIXI, GE Medical Systems, Madison, WI) scans were performed. Mice were exsanguinated via the carotid artery. An aliquot of blood was collected for total white blood cell (WBC) counts and the remainder processed for plasma in EDTA coated tubes and stored at −80°C. All procedures were approved by the Institutional Animal Care and Use Committee of Oklahoma State University.

### 2.2. Intraperitoneal Glucose Tolerance Test

One week prior to the end of the study (23rd wk), mice were fasted for 6 hrs and an intraperitoneal (IP) glucose tolerance test (IGTT) was performed. An IP glucose solution (2 g glucose/kg bodyweight) was administered, followed by blood glucose monitoring at 15, 30, 60, 90, and 120 min. Area under the curve (AUC) was determined by calculating the sum of rectangular area between each time point.

### 2.3. Analysis of Insulin, Adipokines, and Osteocalcin

Plasma insulin was assessed at 4 wk intervals, whereas plasma leptin, adiponectin, and osteocalcin (OCN), both total OCN (Gla-OCN) and undercarboxylated OCN (Glu-OCN), were determined only at the final time point. All assays were performed using commercially available ELISA kits including insulin (Crystal Chem, Downers Grove, IL), leptin and adiponectin (EMD Millipore, Billerica, MA), and Gla-OCN and Glu-OCN (Clontech Takara Bio, Mountain View, CA), following the manufacturer's protocol. Gla-OCN is reported as an indicator of bone turnover and given the importance of the carboxylation status of OCN relative to total OCN on glucose metabolism [20], the ratio of [Glu-OCN]/[Gla-OCN] was calculated to provide insight into the relationship between the skeletal and metabolic response to treatment.

### 2.4. Body Composition and Bone Densitometry

Whole body DXA scans were performed to determine body composition, bone mineral area (BMA), content (BMC), and BMD. All scans were analyzed using PIXImus Series Software version 1.4x (GE Lunar PIXI, Madison, WI).

### 2.5. Microcomputerized Tomography (Micro-CT)

Micro-CT (micro-CT 40, SCANCO Medical, Switzerland) was used to evaluate bone microarchitecture at the proximal tibia metaphysis, tibia middiaphysis, and 4th lumbar vertebral body. Analysis of trabecular bone was performed at the proximal tibia metaphysis on high resolution scans (2048 × 2048 pixels) and the volume of interest (VOI) included 750 *μ*m of secondary spongiosa. The VOI was analyzed using a threshold of 300, a sigma of 0.7, and support of 1.0. Trabecular bone of the vertebra was assessed on images 80 *μ*m from the dorsal and caudal growth plates at medium resolution (1024 × 1024 pixels) and included only secondary spongiosa. Images generated from the scans of the vertebrae were analyzed at a threshold of 340 and a sigma and support of 1.2 and 2.0, respectively. Trabecular parameters evaluated included trabecular bone volume expressed as a percentage of total volume (BV/TV), trabecular number (Tb.N.), trabecular thickness (Tb.Th.), trabecular separation (Tb.Sp.) connectivity density (ConnDens), and structural model index (SMI).

Cortical bone was evaluated by analyzing a 120 *μ*m section at the mid-diaphysis of the tibia. Assessment of cortical bone parameters included cortical porosity, thickness, area, and medullary area of the tibial middiaphysis. The acquired images were analyzed at a threshold of 300, a sigma of 0.7, and support of 1.0.

### 2.6. Analysis of Biomechanical Properties of the Tibia

Tibiae were cleaned of soft-adhering tissue and stored in phosphate buffered saline (PBS) at 4°C until analyses were performed. Reference point indentation (RPI) was applied laterally at the tibia-fibula junction using a BioDent (Active Life Scientific, Inc., Santa Barbara, CA), and the first cycle indentation distance and touchdown distance were recorded. Each tibia was subjected to a testing protocol of 2 N force, 2 Hz, and 10 cycles.

### 2.7. Histology of the Liver

Fixed (10% neutral buffered formalin) liver samples were processed and sectioned (5 *μ*m) for staining with hematoxylin and eosin to assess histological changes associated with nonalcoholic fatty liver disease (NAFLD) that occurs in obesity and/or diabetes. Steatosis and fibrosis were scored on a scale from 0 to 4, with 0 indicating the absence of hepatic lipid droplets or fibrosis, whereas 4 indicated pronounced steatosis or fibrosis. Lobular and portal inflammation was scored using a range of 0–3, with 0 indicating the absence of macrophage infiltration and 3 corresponding to severe inflammation. Balloon degeneration was scored using a 0–2 system, with 0 defined as the lack of degeneration and 2 indicating modest presence of parenchymal cell death. All scoring was performed by the study pathologist who was blinded to treatments.

### 2.8. RNA Isolation and Gene Expression Analysis

Total RNA was isolated from the liver and bone marrow using TriZol Reagent (Invitrogen, Grand Island, NY) as previously described [36, 37]. cDNA was synthesized following a standardized laboratory protocol and qPCR was performed using SYBR green chemistry (7900HT Fast Real-Time, Applied Biosystems, Foster City, CA). Hepatic genes of interest included fatty acid synthase (*Fasn*), sterol regulatory element-binding protein (*Srebp1c*), glucose transporter 2 or solute carrier family (*Slc2a2*), peroxisome proliferative-activator *α* (*Ppara*), and glutathione peroxidase (*Gpx1*) and in the bone marrow* Fasn, Ppara, and Gpx1* (Table S1 available online at http://dx.doi.org/10.1155/2015/758080). All qPCR results were evaluated by the comparative cycle number at threshold (C_Q_) method (User Manual #2, Applied Biosystems) using peptidylprolyl isomerase B or cyclophilin B (*Ppib*) as the invariant control.

### 2.9. Statistical Analysis

Statistical analyses were performed using Statistical Analysis Software version 9.3 (SAS Institute, NC). The primary objective was to determine the difference in response to a HF diet of a given strain and, therefore, Student's paired *t*-test was used unless stated otherwise. However, to further assess differences in the responsiveness between the two C57BL/6 substrains, if a statistical difference (*P* < 0.05) was observed for a given parameter between Con and HF within a given strain, the magnitude of response (i.e., percent change of HF compared to Con) was compared between strains using one-way ANOVA. When the *F* value was <0.05,* post hoc* analyses were performed with Fischer's least square means separation test. Chi-squared tests were used for histological scoring of liver specimens. All data are presented as mean ± standard error (SE) and a *P* < 0.05 was considered statistically significant.

## 3. Results

### 3.1. Body and Fat Pad Weight, Body Composition, and Feed Efficiency

**Figure 1:**
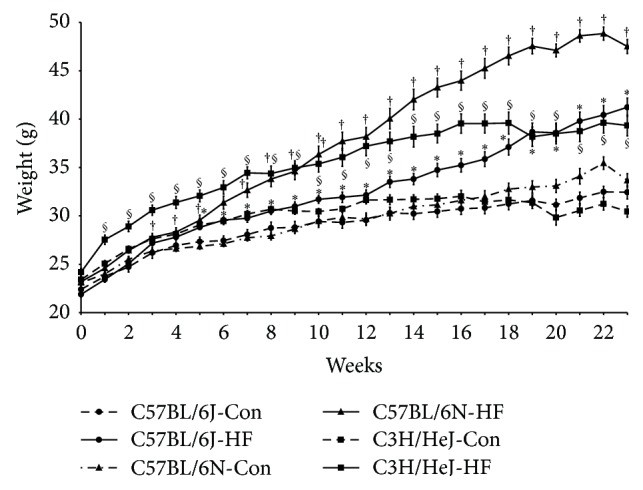
Body weights of C57BL/6J, C57BL/6N, and C3H/HeJ mice fed a control (Con; AIN-93M) or a high fat diet (HF; 45% kcal from fat) were recorded weekly. Data is presented as the mean ± SE, *n* = 15 mice in each group. Symbols, *∗* for C57BL/6J, † for C57BL/6N, or § for C3H/HeJ, indicate significant differences (*P* < 0.05) of dietary treatment within a given mouse strain.

At baseline, body weight between strains differed (C3H/HeJ > C57BL/6N > C57BL/6J); however, no differences existed within a given strain between the two dietary treatment groups (i.e., Con versus HF;* data not shown*). After 5 wk on the HF diet, the C57BL/6J exhibited a significant increase in body weight compared to the C57BL/6J Con, whereas the C57BL/6N on the HF diet had a higher (*P* < 0.05) body weight after only 3 wk (Figure 1). The C3H/HeJ mice on the HF diet also exhibited a more rapid increase in body weight after only 1 wk compared to their respective controls (Figure 1). Analysis of body composition revealed the increase in body weight was due to a significant increase in both lean and fat mass for the two C57BL/6 substrains as well as the C3H/HeJ mice (Table 1). The amount of food consumed was less for the mice on the HF diet in each strain (Table 1). However, on a kcal basis the C57BL/6N mice on the HF diet consumed +2.1 kcal/day and the C57BL/6J and C3H/HeJ on the HF diet consumed +1.2 kcal/day compared to their respective controls (*data not shown*). Overall, feed efficiency was higher in the C57BL/6N mice than in the C57BL/6J mice on the HF diet (Table 1), further demonstrating the differences in metabolic responsiveness between these two substrains.

### 3.2. Tissue Weights and White Blood Cells

After 24 wk on a HF diet the C57BL/6N mice exhibited splenomegaly, thymic hypertrophy, and decreased WBC, but the C57BL/6J mice failed to demonstrate these immunological changes (Table 1). C3H/HeJ mice had a similar response to the HF diet in terms of tissue weights (i.e., spleen and thymus) and total WBCs compared to the C57BL/6N mice (Table 1).

### 3.3. Blood Glucose, Plasma Insulin, and Glucose Tolerance Test

**Figure 2:**
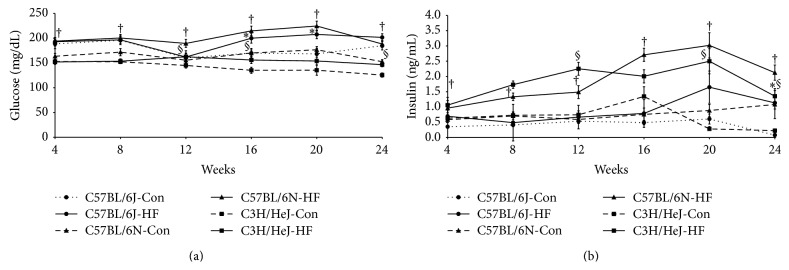
Blood glucose (a) and plasma insulin (b) were determined at 4 wk intervals in mice from each of the three strains fed a control (Con; AIN-93M) or a high fat diet (HF; 45% kcal from fat). Symbols, *∗* for C57BL/6J, † for C57BL/6N, or § for C3H/HeJ, indicate significant differences (*P* < 0.05) of dietary treatment for a given mouse strain.

C57BL/6N mice on the HF diet were the only strain that had elevated fasting blood glucose (Figure 2(a)) and plasma insulin (Figure 2(b)) after 4, 8, 12, 16, 20, and 24 wk of treatment compared to their Con counterparts. The C57BL/6J substrain on the HF diet was hyperglycemic at 16 and 20 wk (Figure 2(a)) and hyperinsulinemic at 24 wk (Figure 2(b)). Importantly, neither substrain achieved a fasting blood glucose consistent with frank diabetes (i.e., >250 mg/dL) that is associated with polyuria and polydipsia [38]. Similar to the C57BL/6J mice, the C3H/HeJ strain on the HF diet exhibited delayed-onset of hyperglycemia (Figure 2(a)), while their plasma insulin was increased at 12, 20, and 24 wk (Figure 2(b)).

**Figure 3:**
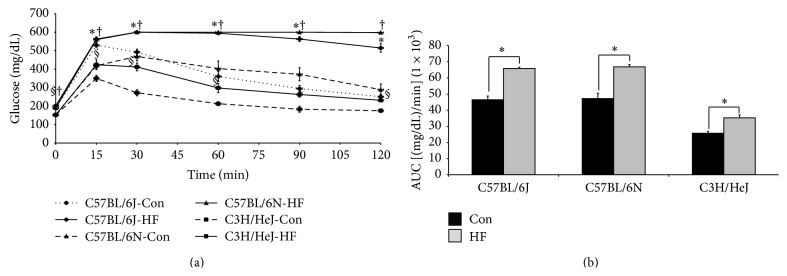
Glucose tolerance test results following 24 wk on control or high fat diet. One week prior to the end of the study, an intraperitoneal glucose tolerance test was administered (2 g glucose/kg body weight) in the C57BL/6J, C57BL/6N, and C3H/HeJ mice fed a control (Con; AIN-93M) or a high fat diet (HF; 45% kcal from fat). (a) Tail blood was collected following 15, 30, 60, 90, and 120 min following glucose injection and symbols, *∗* for C57BL/6J, † for C57BL/6N, or § C3H/HeJ, indicate significant differences (*P* < 0.05) of dietary treatment for a given mouse strain. (b) Area under the curve (AUC) was calculated for the IGTT and symbol, *∗*, represents a significant difference (*P* < 0.05) between dietary treatments for a given strain.

At the end of the study, IGTT showed that the C57BL/6J and C57BL/6N as well as the C3H/HeJ mice on the HF diet exhibited impaired glucose intolerance (Figures 3(a) and 3(b)). The percent change in AUC to the HF diet demonstrated that the magnitude of response of the two C57BL/6 substrains was similar (*data not shown*). The C3H/HeJ mice also exhibited impaired glucose intolerance after 24 wk on a HF diet (Figure 3). It should be noted that, despite elevated AUC, the C3H/HeJ mice on the HF diet maintained the ability to restore blood glucose by the final IGTT time point.

### 3.4. Plasma Adipokines and Osteocalcin

Both the C57BL/6J and C57BL/6N substrains had elevated plasma leptin after 24 wk on a HF diet (Table 1), but there was no significant difference in the magnitude of the response between the two substrains. Similarly, the C3H/HeJ mice on the HF diet also had higher plasma leptin (Table 1). Interestingly, at 24 wk the C57BL/6J mice, but not the C57BL/6N substrain, exhibited a decrease in plasma adiponectin in response to a HF diet (Table 1).

**Figure 4:**
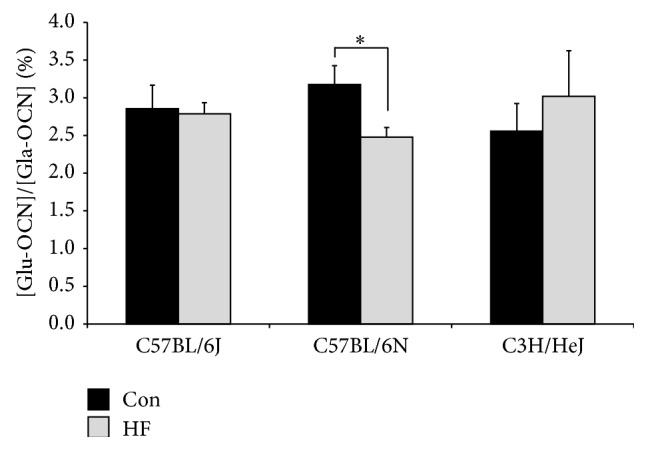
Plasma osteocalcin (OCN) expressed as percent of undercarboxylated Glu-OCN per total Gla-OCN in C57BL/6J, C57BL/6N, and C3H/HeJ mice on a control (Con; AIN-93M) or a high fat diet (HF; 45% kcal from fat) after 24 wk. Symbol, *∗*, represents a significant difference (*P* < 0.05) between dietary treatments for a given strain.

After 24 wk on a HF diet, there were no differences in Gla-OCN as an indicator of bone turnover due to diet in the C57BL/6 substrains or C3H/HeJ mice (*data not show*). The carboxylation status of OCN (i.e., Glu/Gla-OCN ratio), which has been shown to influence insulin sensitivity and systemic energy metabolism, was reduced only in the C57BL/6N mice after 24 wk on a HF diet (Figure 4).

### 3.5. Histological Evaluation of Hepatic Tissue

**Figure 5:**
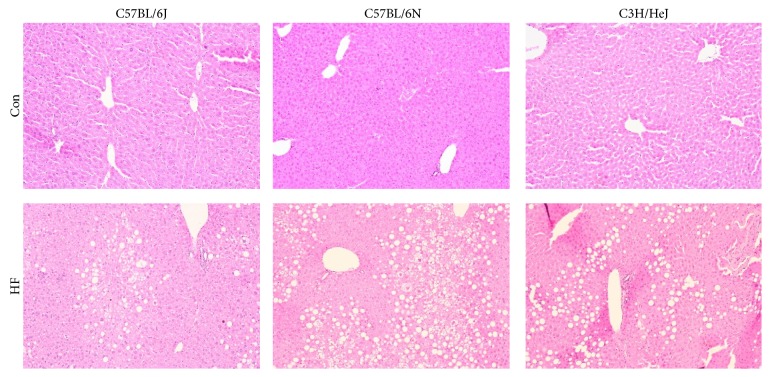
Representative micrographs of liver histology sections stained with hematoxylin and eosin from C57BL/6J, C57BL/6N, and C3H/HeJ mice following 24 wk on a control (Con; AIN-93M) or a high fat diet (HF; 45% kcal from fat). Representative images were photographed and are presented at a 10x magnification.

Representative micrographs of liver sections from each group show that the C57BL/6J and C57BL/6N strains as well as the C3H/HeJ strain experienced some degree of hepatic steatosis in response to the HF diet (Figure 5). The C57BL/6N mice on the HF diet had a significantly higher lobular and portal inflammation mean score compared to the Con (Table 2). Although the C57BL/6J mice on the HF diet had more lobular inflammation than their respective controls (*P* = 0.0038), the frequency of the inflammatory response was markedly lower in this substrain compared to the C57BL/6N (i.e., lobular inflammation in 92% C57BL/6N versus 54% C57BL/6J and portal inflammation in 77% C57BL/6N versus 23% C57BL/6J) (Table 2). While none of the C57BL/6J mice on the HF diet exhibited liver fibrosis, 23% of the treated C57BL/6N mice had fibrotic changes (Table 2). Balloon degeneration was also more severe in the C57BL/6N mice on the HF diet compared to the C57BL/6J (Table 2). Despite a lack of lobular and portal inflammation and fibrosis in the C3H/HeJ mice, balloon degeneration was severe in this strain (Table 2).

### 3.6. Whole Body Bone Densitometry

Both the C57BL/6J and C57BL/6N mice demonstrated a decrease in whole body BMC and BMA, but no change in whole body BMD in response to the HF diet after 24 wk (Table 3). When BMD was expressed relative to body weight, differences due to diet were observed suggesting that the bone density did not increase relative to the increase in body weight (Table 3).

### 3.7. Microarchitectural Changes in Trabecular and Cortical Bone

**Figure 6:**
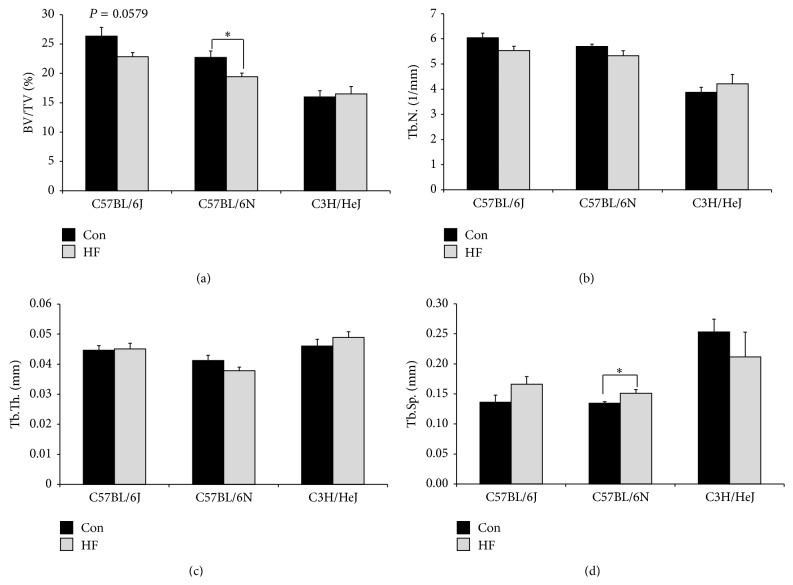
Micro-CT analyses of trabecular bone in the lumbar vertebra (L4) in C57BL/6J, C57BL/6N, and C3H/HeJ mice on a control (Con; AIN-93M) or a high fat diet (HF; 45% kcal from fat) for 24 wk. Parameters include (a) bone volume/total volume (BV/TV), (b) trabecular number (Tb.N.), (c) trabecular thickness (Tb.Th.), and (d) trabecular separation (Tb.Sp.). Symbol, *∗*, represents a significant difference (*P* < 0.05) between dietary treatments for a given strain.

Micro-CT analyses of the lumbar vertebra revealed significant loss of trabecular bone or BV/TV with the HF diet in C57BL/6N, while the skeletal response of the C57BL/6J mice did not reach the level of statistical significance (*P* < 0.0579) (Figure 6(a)). As expected, the C3H/HeJ mice were protected from vertebral bone loss (Figure 6) or nonmorphometric parameters with HF diet (Table 3). Both the C57BL/6J and C57BL/6N mice on the HF diet had a higher SMI indicative of a weaker, more rod-like trabecular bone in the vertebra (Table 3).

In contrast to the vertebra, no changes were observed in trabecular or cortical parameters analyzed at the proximal tibial metaphysis or the tibial middiaphysis in the C57BL/6J or the C57BL/6N mice. The C3H/HeJ mice failed to demonstrate alterations in trabecular bone of the proximal tibia but did exhibit an increase in the medullary area at the middiaphysis (Table 3).

### 3.8. Changes in Biomechanical Properties of the Tibia

Based on reference point indentation testing on cortical bone at the tibia-fibula junction, no changes were observed in first cycle indentation distance or touchdown distance in any strain following 24 wk on a HF diet when compared to their respective Con (Table 3).

### 3.9. Characterization of Genes Involved in Energy Metabolism and Inflammation from the Liver and Bone Marrow

Determination of genes involved in hepatic metabolism and inflammation revealed that the C57BL/6N mice on the HF diet had altered metabolic processes, including the upregulation of glucose uptake (*Slc2a2*), triglyceride storage (*Fasn* and* Srebp1c*) and adipogenesis (*Ppara*), as well as antioxidant capacity (*Gpx1*) (Table 4). Interestingly, none of these alterations in gene expression were observed in the C57BL/6J mice after 24 wk on the HF diet.

To determine the degree to which oxidative stress and adipogenesis contributed to bone loss with the HF diet model,* Gpx1* and* Pparg* mRNA abundance was determined in the bone marrow. Similar to the hepatic tissue, the abundance of* Gpx1* mRNA was increased in the C57BL/6N mice on the HF diet, suggesting an increase in antioxidant capacity (Table 4). In contrast, the C57BL/6J mice on the HF diet demonstrated a decrease in the relative abundance of* Gpx1* (Table 4). Additionally, no alterations were observed in the transcriptional regulator of adipogenesis,* Pparg*, in any strain after 24 wk (Table 4).

## 4. Conclusions

The findings of this study show that the C57BL/6J and the C57BL/6N mouse differ in their metabolic response to a HF diet over a 24 wk study period. Discrepancies in the metabolic response between the two strains may be attributed in part to the missense mutation (M35T) in exon 1 and a multiexon deletion of* Nnt* in the C57BL/6J mice [39, 40]. This mutation in* Nnt* has been reported to uncouple *β*-cell mitochondrial metabolism leading to less ATP production in pancreatic islets, enhanced K_ATP_ channel activity, and, consequently, impaired glucose-stimulated insulin secretion [23, 39, 41]. Only fasting insulin was assessed in the current study; however, early onset of hyperinsulinemia with HF diet was only observed in the C57BL/6N mice with an intact, functional* Nnt*. The coincident lower feed efficiency in the C57BL/6J compared to the C57BL/6N mice resulted in a delay in the development of hyperglycemia and shorter duration of exposure to conditions associated with impaired glucose tolerance.

The C57BL/6J and C57BL/6N mice had a markedly different hepatic response to the HF diet after 24 wk. Increased mRNA abundance of* Fasn* and a modest increase in* Srebpc1* in the presence of severe liver steatosis in the C57BL/6N mice on the HF diet suggest an increase in hepatic triglyceride synthesis and storage. Conversely, the C57BL/6J substrain, which has lower glucokinase activity and thus impaired glucose sensing, may explain the lack of transcriptional regulation of* Fasn* and* Srebp1c* [39]. Furthermore, C57BL/6N mice on the HF diet demonstrated an increase in* Slc2a2* gene expression, which encodes the non-insulin-sensitive glucose transporter 2 and has been reported to be upregulated in response to a HF diet [42]. Histological evaluation suggests that the C57BL/6N mice on the HF diet also experienced the most pronounced hepatic inflammation, compared to the C57BL/6J mice. Interestingly, the HF-induced NAFLD that develops in the C57BL/6J was previously attributed, in part, to the spontaneous mutation in the* Nnt* [39, 43]; however, the data demonstrate that the C57BL/6N mice, with a functional* Nnt*, develop more severe NAFLD in response to HF diet feeding. Given the more pronounced metabolic phenotype in the C57BL/6N mice on the HF diet, it was counterintuitive that only the C57BL/6J mice demonstrated the anticipated decrease in plasma adiponectin following a HF diet. Although the mechanism for reduced adiponectin during obesity and T2DM has been attributed, in part, to an increase in local TNF-*α* in adipose tissue [44], strain-related variability of adiponectin gene expression and plasma has been previously documented [45]. As mice continue to be used for models of impaired glucose homeostasis, further investigation is warranted to fully understand these strain differences relative to metabolic handling. The findings of this study also demonstrate that the C3H/HeJ mice may not be completely resistant to diet-induced obesity and the subsequent metabolic changes. Differences in the C3H/HeJ strain's response to a high fat diet compared to previous reports may be attributed to the difference in the control strain used [26, 28]. Specifically, previous studies have compared the C3H/H3J response to HF diet to C3H/HeOuJ or C3H/HeN, both of which have a functional TLR-4 [26, 28]. However, in these studies, no comparisons were made with C3H/HeJ mice on a control diet. Therefore, it is not possible to determine if the differences in metabolic response to a HF diet are a result of TLR-4 or genetic variability in the control substrain background. Furthermore, the C3H/HeJ strain has recently been shown to have a genetic variation in the leptin receptor gene (*Lepr*) [46]. This mutation could account for the impaired metabolic response observed in the C3H/HeJ strain on a HF diet due to the central role leptin has on regulating energy intake and expenditure. However, in the present study, food intake in the C3H/HeJ strain was not significantly altered and the absence of a leptin-mediated effect on food or energy intake does not rule out implications of the* Lepr* defect on the skeletal response [18, 19, 47].

In conjunction with the metabolic comparisons, the other primary objective of this study was to compare the skeletal response to a HF diet in two commonly used C57BL/6 substrains. The C57BL/6J mice on the HF diet experienced a 13.6% reduction in trabecular bone of the vertebra, although not statistically significant. C57BL/6N was the only strain that exhibited significant trabecular bone loss which occurred only in the vertebra. In the absence of alterations in tibia trabecular and cortical bone microarchitecture, it is conceivable that the absence of alterations in the tibia could result from site-specific changes associated with increased adiposity and greater weight-bearing, which could offset some of the negative effects of glucose intolerance on bone [48, 49]. Based on reports in the literature that bone biomechanical properties are changed in T2DM independent of alterations in bone mass [8, 50, 51], the tibia was subjected to RPI testing. No detectable alterations in cortical bone strength were observed after 6 months in the absence of structural changes. The C57BL/6N mice had more prolonged exposure to hyperglycemia and hyperinsulinemia in response to the HF diet compared to the C57BL/6J substrain, and C57BL/6N were the only substrain to lose significant trabecular bone in the spine. While there have been conflicting reports on how a HF diet impacts bone in C57BL/6 mice [12, 14–17], the results of this study indicate the skeletal response may be linked to the duration of disrupted insulin signaling and glucose intolerance. This idea is further supported by the response of the C3H/HeJ mice in which case an attenuated glucose, leptin, and insulin response to the HF diet failed to induce bone loss. Several reports have shown that a high fat diet uncouples bone turnover by increasing bone resorption and decreasing bone formation in various rodent models [16, 17, 21]. In this study, osteocalcin which is considered a marker of bone turnover was the only bone marker assessed and it was not altered at the end of the study. Although this does not rule out alterations in bone metabolism occurring earlier, future studies are needed to investigate the mechanism involved in the site-specific loss of bone observed in this animal model.

Based on recent literature describing the hormone OCN as a regulator of systemic energy metabolism [52–54], the role of OCN on both metabolic and skeletal changes induced by HF was investigated. After 24 wk, the C57BL/6N mice on the HF diet had a lower ratio of plasma Glu-OCN/Gla-OCN. Because circulating undercarboxylated (Glu-OCN) can act directly on pancreatic *β*-cells to stimulate insulin secretion [20, 55–57], it would be expected that a reduction in Glu-OCN would lead to a decrease in insulin secretion. Instead, fasting plasma insulin was elevated in the C57BL/6N mice on the HF diet compared to their respective controls. Alternatively, these results could indicate the direct effects impaired glucose tolerance has on osteoclastogenesis and bone resorption [58]. In this regard, the acidic milieu of the osteoclast-resorption lacunae is capable of decarboxylating OCN, resulting in its release from the bone matrix and its subsequent circulation in the blood [55]. The complexity of OCN's role on bone and energy metabolism during glucose intolerance, as well as these implications in various mouse strains, warrants further investigation. Additional studies are also needed to determine how genes involved in the gamma carboxylation of OCN (i.e.,* Ggcx* and* Esp1*) by osteoblasts as well as the role of decarboxylation of OCN by osteoclast are regulated in response to changes energy homeostasis [55, 59].

To date, this is the first study to directly compare the C57BL/6J and C57BL/6N substrains' response to a HF diet from a metabolic and skeletal perspective. Although neither substrain developed frank T2DM, the data presented here show that C57BL/6N mice exhibit an earlier metabolic response consistent with impaired glucose tolerance or prediabetes to the HF diet compared to the C57BL/6J mice. Moreover, the skeletal response followed that of the metabolic changes; this was demonstrated by the fact that significant trabecular bone loss occurred in the C57BL/6N mice, which demonstrated the robust metabolic alterations associated with clinical T2DM. Given the observed differences in feed efficiency between the C57BL/6 substrains, further research is also warranted to identify the mechanisms underlying altered energy utilization. In contrast to the C57BL/6 mice, the C3H/HeJ strain was protected from the metabolic and skeletal changes induced by a HF diet. While a number of questions remain including how bone metabolism is being altered in response to a high fat diet on a molecular level, this study highlights the need to consider not only the most appropriate strain but also the most appropriate substrain of mouse when designing experiments. Other important factors to consider when studying the relationship between glucose intolerance and bone include the site-specific skeletal response and the study duration. These decisions could significantly impact data interpretation and the translational implications as they relate to understanding how bone metabolism is altered in the context of T2DM.

## Supplementary Material

Supplemental Table 1. Accession numbers and nucleotide sequences for the forward (F) and reverse (R) primer sets used for qPCR.

## Figures and Tables

**Table 1:** Body composition, tissue weights, food intake, total white blood cell counts, and adipokines.

	C57BL/6J			C57BL/6N			C3H/HeJ		
	Con	HF	*P* values	Con	HF	*P* values	Con	HF	*P* values
Final body weight (g)	32.4 ± 0.7	41.2 ± 0.9	***<0.0001***	33.7 ± 0.7	47.5 ± 0.7	***<0.0001***	30.5 ± 0.6	39.3 ± 1.1	***<0.0001***
Body composition									
Lean (g)	21.8 ± 0.4	24.9 ± 0.3	***<0.0001***	22.1 ± 0.3	27.7 ± 0.5	***<0.0001***	19.8 ± 0.3	23.9 ± 0.5	***<0.0001***
Fat (g)	7.1 ± 0.4	12.0 ± 1.2	***0.0006***	9.6 ± 0.6	18.3 ± 0.6	***<0.0001***	6.7 ± 0.3	11.1 ± 0.6	***<0.0001***
Percent fat (%)	24.2 ± 1.0	31.8 ± 1.4	***0.0018***	29.4 ± 1.2	39.7 ± 0.9	***<0.0001***	25.2 ± 0.8	31.5 ± 0.8	***<0.0001***
Fat pad (g)	0.68 ± 0.08	1.67 ± 0.13	***<0.0001***	1.14 ± 0.11	1.69 ± 0.07	***0.0067***	0.52 ± 0.07	0.90 ± 0.06	***0.0051***
Feed efficiency (mg/kcal)	4.63 ± 0.38	8.41 ± 0.43	***0.0011***	5.28 ± 0.34	11.01 ± 0.43	***<0.0001***	3.24 ± 0.26	6.17 ± 0.36	***0.0086***
Food intake (g/day)	3.17 ± 0.04	2.74 ± 0.06	***<0.0001***	3.22 ± 0.04	2.98 ± 0.04	***0.0002***	3.19 ± 0.04	2.78 ± 0.06	***<0.0001***
Thymus (g)	0.042 ± 0.002	0.048 ± 0.003	*0.1639 *	0.055 ± 0.004	0.076 ± 0.006	***0.0096***	0.019 ± 0.002	0.024 ± 0.002	*0.0984 *
Spleen (g)	0.091 ± 0.005	0.098 ± 0.004	*0.2754 *	0.106 ± 0.006	0.132 ± 0.009	***0.0323***	0.101 ± 0.004	0.123 ± 0.006	***0.0066***
WBC (1 × 10^5^)	17.04 ± 1.80	18.55 ± 1.61	*0.5350 *	21.69 ± 1.55	16.14 ± 1.72	***0.0045***	7.60 ± 0.71	11.48 ± 1.05	***0.0252***
Adipokines									
Leptin (ng/mL)	3.64 ± 1.14	16.13 ± 3.67	***0.0045***	8.92 ± 1.87	44.96 ± 5.06	***<0.0001***	0.84 ± 0.15	11.08 ± 2.72	***0.0014***
Adiponectin (*μ*g/mL)	9.94 ± 0.76	7.45 ± 0.72	***0.0068***	6.36 ± 0.34	6.97 ± 0.25	*0.1637 *	5.05 ± 0.40	5.13 ± 0.17	*0.8315 *

Final body weight, body composition, food intake, tissue weight, and total white blood cell counts (WBC) and adipokines after 24 wk on a control (Con = AIN-93M) or a high fat diet (HF = 45% kcal from fat) in C57BL/6J, C57BL/6N, and C3H/HeJ mice. Values are means ± SE, *n* = 15 mice in each group. *P* values show the differences between dietary treatments within a given strain.

**Table 2:** Pathological scoring of hepatic tissue after 24 wk on a control or high fat diet.

	C57BL/6J							C57BL/6N							C3H/HeJ						
	0	1	2	3	4	Mean	*P* value	0	1	2	3	4	Mean	*P* value	0	1	2	3	4	Mean	*P* value
Steatosis																					
Con	10	1	0	0	0	0.09	***0.0028***	6	2	3	0	0	0.73	***0.0020***	10	0	0	0	0	0.00	***0.0001***
HF	2	4	6	1	0	1.46		0	2	1	2	8	3.23		1	4	5	4	0	1.86	
Fibrosis																					
Con	0	0	0	0	0	0.00	—	11	0	0	0	0	0.00	*0.0885 *	10	0	0	0	0	0.00	—
HF	0	0	0	0	0	0.00		10	3	0	0	0	0.23		14	0	0	0	0	0.00	
Lobular inflammation																					
Con	11	0	0	0		0.00	***0.0038***	11	0	0	0		0.00	***<0.0001***	10	0	0	0		0.00	*0.0641 *
HF	6	7	0	0		0.54		1	12	0	0		0.92		10	4	0	0		0.29	
Portal inflammation																					
Con	11	0	0	0		0.00	*0.0885 *	11	0	0	0		0.00	***0.0001***	10	0	0	0		0.00	—
HF	10	3	0	0		0.23		3	10	0	0		0.77		14	0	0	0		0.00	
Balloon degeneration																					
Con	0	0	0			0.00	***0.0062***	11	0	0			0.00	***<0.0001***	10	0	0			0.00	***<0.0001***
HF	5	7	1			0.69		0	0	13			2.00		1	3	10			1.64	

Frequency of steatosis (0–4), lobular and portal inflammation (0–3), fibrosis (0–4), and balloon degeneration (0–2), along with mean scores and *P* values for control versus high fat diet within a given strain based on Chi-squared statistical analyses.

**Table 3:** Bone densitometry of the whole body along with bone microarchitectural and biomechanical parameters of the spine and tibia.

	C57BL/6J			C57BL/6N			C3H/HeJ		
	Con	HF	*P* values	Con	HF	*P* values	Con	HF	*P* values
Whole body bone densitometry									
BMC (mg)	731.2 ± 30.3	622.8 ± 23.6	***0.0101***	614.5 ± 24.1	492.9 ± 11.8	***<0.0001***	878.5 ± 25.1	810.1 ± 14.3	***<0.0001***
BMA (cm^2^)	12.31 ± 0.40	10.87 ± 0.42	***0.0149***	11.03 ± 0.33	8.91 ± 0.12	***<0.0001***	12.90 ± 0.15	11.76 ± 0.22	***<0.0001***
BMD (mg/cm^2^)	59.0 ± 0.8	57.2 ± 0.6	*0.0866 *	55.9 ± 0.8	55.4 ± 0.6	*0.6817 *	69.5 ± 0.7	68.6 ± 0.7	*0.3777 *
BMD/BW [(mg/cm^2^)/g]	1.84 ± 0.05	1.39 ± 0.05	***<0.0001***	1.67 ± 0.04	1.17 ± 0.02	***<0.0001***	2.29 ± 0.05	1.76 ± 0.05	***<0.0001***
Lumbar vertebra trabecular									
Conn density (1/mm^3^)	232.45 ± 13.37	249.77 ± 10.96	*0.3398 *	221.21 ± 14.25	214.62 ± 16.73	*0.7766 *	145.78 ± 18.26	116.81 ± 10.83	*0.2023 *
SMI	0.30 ± 0.09	0.67 ± 0.02	***0.0028***	0.59 ± 0.10	1.00 ± 0.09	***0.0136***	1.10 ± 0.11	1.07 ± 0.09	*0.8508 *
App density (mg HA/ccm)	395.86 ± 20.97	364.38 ± 7.04	*0.1852 *	358.84 ± 7.84	318.54 ± 8.85	***0.0087***	279.49 ± 10.48	287.93 ± 11.99	*0.6070 *
Mat density (mg HA/ccm)	1093.1 ± 6.7	1084.0 ± 8.9	*0.4366 *	1078.5 ± 8.3	1076.9 ± 8.2	*0.9970 *	1123.5 ± 10.2	1135.5 ± 11.1	*0.4464 *
Tibial midshaft									
Cortical porosity (%)	4.30 ± 0.26	4.35 ± 0.13	*0.2026 *	4.50 ± 0.14	4.71 ± 0.10	*0.8831 *	3.07 ± 0.08	3.69 ± 0.45	*0.0505 *
Cortical thickness (mm)	0.17 ± 0.04	0.19 ± 0.04	*0.0796 *	0.14 ± 0.03	0.16 ± 0.03	*0.8602 *	0.18 ± 0.05	0.20 ± 0.04	*0.1381 *
Cortical area (mm^2^)	0.011 ± 0.001	0.010 ± 0.001	*0.0535 *	0.010 ± 0.001	0.011 ± 0.001	*0.8756 *	0.014 ± 0.001	0.013 ± 0.001	*0.3270 *
Medullary area (mm^2^)	0.035 ± 0.001	0.032 ± 0.001	*0.8879 *	0.034 ± 0.004	0.037 ± 0.002	*0.7738 *	0.030 ± 0.001	0.034 ± 0.001	***0.0218***
Indentation distance (*μ*m)	34.7 ± 1.5	32.9 ± 0.9	*0.3231 *	29.9 ± 0.7	34.9 ± 4.5	*0.2603 *	35.3 ± 1.1	38.4 ± 3.1	*0.3587 *
Touchdown distance (*μ*m)	162.1 ± 12.9	150.2 ± 8.2	*0.4465 *	148.2 ± 6.5	179.6 ± 14.7	*0.0615 *	158.8 ± 10.8	157.6 ± 6.7	*0.4269 *

Whole body bone densitometry and trabecular and cortical bone microarchitecture, along with reference point indentation (RPI) after 24 wk on a control (AIN-93M) or a HF (45% kcal from fat). Values are means ± SE, *n* = 10–15 mice per group for bone densitometry and *n* = 6 mice per group for micro-CT data. *P* values show the differences between dietary treatments within a given strain. Values are means ± SE.

**Table 4:** Relative fold change of gene expression in the liver and bone marrow in mice fed a high fat diet compared to the control diet.

	C57BL/6J		C57BL/6N		C3H/HeJ	
	HF	*P* value	HF	*P* value	HF	*P* value
Liver						
*Fasn *	1.55 ± 0.31	*0.1748 *	2.20 ± 0.36^*∗*^	***0.0248***	1.23 ± 0.35	*0.5934 *
*Gpx1 *	0.89 ± 0.12	*0.6197 *	1.54 ± 0.15^*∗*^	***0.0127***	1.31 ± 0.14	*0.1653 *
*Ppara *	1.10 ± 0.16	*0.5427 *	1.41 ± 0.11^*∗*^	***0.0478***	0.98 ± 0.10	*0.8582 *
*Slc2a2 *	0.99 ± 0.10	*0.9792 *	1.56 ± 0.14^*∗*^	***0.0096***	1.08 ± 0.21	*0.7756 *
*Srebp1c *	1.20 ± 0.26	*0.4985 *	2.07 ± 0.51	*0.0997 *	0.93 ± 0.21	*0.8329 *
*Tnf *	1.76 ± 0.40	*0.2237 *	1.12 ± 0.28	*0.7238 *	2.09 ± 0.51	*0.0703 *
Bone marrow						
*Fasn *	0.94 ± 0.05	*0.6811 *	0.94 ± 0.07	*0.5619 *	1.20 ± 0.15	*0.2388 *
*Gpx1 *	0.56 ± 0.08^*∗*^	***0.0053***	1.45 ± 0.13^*∗*^	***0.0228***	1.05 ± 0.18	*0.7983 *
*Pparg *	1.35 ± 0.39	*0.4349 *	0.78 ± 0.10	*0.1829 *	1.21 ± 0.18	*0.3279 *

Mean fold regulation of genes involved in systemic metabolism and inflammation is presented for the animals on the high fat diet (45% kcal from fat) relative to their control (AIN-93M). All target genes were normalized to invariant control (*Ppib*). Symbol, *∗*, represents a significant difference (*P* = 0.05) between dietary treatments within a given strain.
