# Supplementary material for: A Comparative Study of the Metabolic and Skeletal Response of C57BL/6J and C57BL/6N Mice in a Diet-Induced Model of Type 2 Diabetes
Source: J Nutr Metab. 2015 Jun 3;2015:758080. doi: 10.1155/2015/758080 (PMC4469802; doi:10.1155/2015/758080)
Supplement: Supplementary file 1 — Supplemental Table 1. Accession numbers and nucleotide sequences for the forward (F) and reverse (R) primer sets used for qPCR. [file 758080.f1.docx]

| **NCBI Gene Accession Reference** | **Symbol** | **Name** | **Sequence** |
| --- | --- | --- | --- |
| NM_007988.3 | *Fasn* | Fatty acid synthase | F 5'- GCT GCG GAA ACT TCA GGA AAT -3' |
|  |  |  | R 5'- AGA GAC GTG TCA CTC CTG GAC TT -3' |
| NM_008160.6 | *Gpx1* | Glutathione peroxidase | F 5'- CGG TTT CCC GTG CAA TC -3' |
|  |  |  | R 5'- GAG GGA ATT CAG AAT CTC TTC AT -3' |
| XM_006520619.1 | *Ppara* | Peroxisome proliferator activated receptor alpha | F 5'- CGT ACG GCA ATG GCT TTA TC -3' |
|  |  |  | R 5'- AAC GGC TTC CTC AGG TTC TT -3' |
| XM_006505737.1 | *Pparg* | Peroxisome proliferator activated receptor gamma | F 5'- CAA GAA TAC CAA AGT GCG ATC AA -3' |
|  |  |  | R 5'- GAG CTG GGT CTT TTC AGA ATA ATA AG -3' |
| NM_011149.2 | *Ppib* | Peptidylprolyl isomerase B | F 5'- TGG AGA GCA CCA AGA CAG ACA -3' |
|  |  |  | R 5'- TGC CGG AGT CGA CAA TGA T -3' |
| NM_031197.2 | *Slc2a2* | Solute carrier family 2 (facilitated glucose transporter), member 2 | F 5'- CAA CTG GGT CTG CAA TTT TGT C -3' |
|  |  |  | R 5'- GAA CAC GTA AGG CCC AAG GA -3' |
| XM_006532716.1 | *Srebp1c* | Sterol regulatory element-binding protein | F 5'- GGA GCC ATG GAT TGC ACA TT -3' |
|  |  |  | R 5'- GGC CCG GGA AGT CAC TGT -3' |
| NM_001278601.1 | *Tnf* | Tumor necrosis factor | F 5'- CTG AGG TCA ATC TGC CCA AGT AC -3' |
|  |  |  | R 5'- CTT CAC AGA GCA ATG ACT CCA AAG -3' |

Supplemental Table 1. qPCR Primer List
